# Stabilization Techniques of Essential Oils by Incorporation into Biodegradable Polymeric Materials for Food Packaging

**DOI:** 10.3390/molecules26206307

**Published:** 2021-10-19

**Authors:** Elena Stoleru, Mihai Brebu

**Affiliations:** Laboratory of Physical Chemistry of Polymers, “Petru Poni” Institute of Macromolecular Chemistry, 41A Gr. Ghica Voda Alley, 700487 Iaşi, Romania

**Keywords:** bioactive packaging, herbal extracts, food safety, emulsification, electrospinning

## Abstract

Human health, food spoilage, and plastic waste, which are three great topical concerns, intersect in the field of food packaging. This has created a trend to replace synthetic food preservatives with natural ones, to produce bio-functional food packaging, and to shift towards biodegradable polymeric materials. Among the natural bioactive agents, essential oils are gaining more and more attention in food packaging applications due to their various benefits and fewer side-effects. However, their volatility, hydrophobicity, and strong odor and taste limit the direct use in food-related applications. Fixation into polymeric matrices represents a suitable strategy to promote the benefits and reduce the drawbacks. Emulsification and electrospinning are largely used techniques for protection and stabilization of essential oils. These methods offer various advantages in active food packaging, such as controlled release, ensuring long-term performance, decreased amounts of active agents that gain enhanced functionality through increased available surface area in contact with food, and versatility in packaging design. This review focuses on creating correlations between the use of essential oils as natural additives, stabilization methods, and biodegradable polymeric matrices or substrates in developing bioactive food packaging materials. Documentation was performed via the Scopus, ScienceDirect, and PubMed databases, selecting the publications after the year 2018. Particular attention was given to the publications that tested materials on food/food pathogens to evaluate their performances in retarding spoilage. Research gaps were also identified on the topic, materials being tested mainly at short time after preparation without considering the long-term storage that usually occurs in actual practice between production and use, and insufficient research related to upscaling.

## 1. Introduction

Packaging occupies an increasingly important place in the food industry, having several roles in storage and transportation, since most food is not immediately consumed after production. The conventional assignments of food packaging are maintaining physical integrity, sanitary, safety, and quality features. Moreover, it should ensure protection against various environmental factors such as UV radiation, oxygen, humidity, and contaminants [1]. Nowadays packaging has to keep pace with the continuous change of lifestyle that involves increasing diversification of food (e.g., modern and alien foods competing with traditional and indigenous ones, increased varieties of food) and modification of nutritional habits (e.g., fast food versus slow food). Huge amounts of food are wasted yearly, reaching almost one-third of production [2]. Food spoilage occurs mainly under microbiological action that significantly decreases the shelf-life of foodstuffs and can negatively impact human health if not properly addressed. Oxidation is also a common process that reduces food quality. This induces alteration of flavor and color, vitamin degradation, and lipid rancidity. Therefore, the new trend in fighting against food spoilage is to include antimicrobial and antioxidant agents in food packaging materials. This is of great help in the current search for strategies to increase food products’ overall quality and storage period [3]. Among these, active packaging attracts strong interest from researchers and industry due to its potential to provide food quality and safety benefits, being considered a modern short-term preservation method. These materials strongly differ from the traditional, generally inert packaging materials, by their interactions with the surrounding medium or with food and its headspace through controlled release of functional substances that can extend the shelf-life of the products [4,5].

Research on active food packaging records a highly increasing trend reflected in a large number of recent publications. Although numerous reviews are reported in the scientific literature on the topic of the use of natural bioactive agents in active food packaging, at the moment there is a lack of correlation between the incorporation methods, the type of matrices, and the functions of the active compounds. This review highlights the possibilities and limitations of the current approaches for incorporation of bioactive compounds into food packaging materials and tries to correlate the functionalities of active agents iwith the properties of the polymeric matrix/substrate. The primaryinterest is focused here on the two main methods for fixation of bioactive compounds into polymeric matrices, namely emulsification and electrospinning, limiting the area of discussions to essential oils and biodegradable matrices. This follows the current trend of replacing synthetic food preservatives with natural ones, imparting bioactivity to packaging materials and shifting toward polymers with adequate biodegradability. Scopus, ScienceDirect, and PubMed databases were used for documentation, considering original research articles and reviews published after the year 2018 as inclusion and exclusion criteria. Older relevant publications in the field were also included, especially when dealing with fundamental aspects on the discussed topic (e.g., classification of emulsions or the electrospinning concept).

## 2. Generalities on Active Food Packaging

Classical preservation methods involve use of synthetic chemical compounds that perturb the evolution of food spoilage microorganisms or inhibit oxidative processes that reduce freshness and decrease the nutritional value of food. However, most chemical preservatives have negative effects on human health, such as intolerance or allergy to foods, nausea, attention deficit or hyperactivity disorder, cardiac diseases, etc., which are increasing consumer concerns [6,7]. Carcinogenicity and disruption effects on thyroid hormone functionality were reported for nitrates and nitrites, which are largely used as preservatives and color enhancers in meats [8]. A great demand was noted in recent decades to replace synthetic preservatives with natural additives, whose main properties of interest are antimicrobial and antioxidant activity.

Most natural bioactive agents in food packaging are either low molecular or high molecular compounds that originate from vegetal materials, some of them being obtained from by-products of fruit and plant processing or from underexploited plant species [9]. For example, plant extracts, essential (EO) and vegetal (VO) oils, and aromatic herbs have been demonstrated as efficient antimicrobial and antioxidant agents. This is due to the presence in their composition of beneficial phytochemicals such as terpenoids, phenols and derivatives, flavonoids, coumarins, quinines, saponins, tannins, and alkaloids [10]. Since these compounds are produced by plant metabolism, especially in direct relation with pollinating insects or threatening microorganisms, they usually have low toxicity to mammals and humans, which are not considered target species for defense [11].

However, the numerous beneficial properties of natural additives come with the expense of their low stability under the action of environmental factors such as temperature, oxidative agents, and UV radiation. Therefore, they have to be protected and stabilized against aggressive factors to which materials could be exposed during their processing or utilization. For example, highly volatile essential oils, which are increasingly considered for applications in food packaging [12], can undergo volatilization during thermal processing [13]. On the other hand, natural additives with strong odor and taste could affect the organoleptic characteristics of food [14,15]. Hence, protection of food from the negative impact of additives should be also considered [16]. Both aspects could be addressed by encapsulation into matrices, which also increases the effective surface area and, consequently, the activity [17]. The presence of bioactive compounds in packaging is more important at the surface of the material, which is in direct contact with food surface where spoilage or microbial growth initiates [18]. This can be obtained by surface functionalization or by ensuring optimum migration from bulk to surface [19].

Among natural bioactive agents, vegetal and essential oils have gained increased attention due to their numerous functional properties suitable for food-related applications, mainly antimicrobial activity and antioxidant or oxygen scavenging potential [20]. Essential oils are plant secondary metabolites which are thought to be safer than synthetic chemical preservatives [21]. They are generally recognized as safe (GRAS) and approved by the U.S. Food and Drug Administration (FDA) [22], therefore suitable for use in food-related applications without the need for approval [23]. Due to their hydrophobic nature, essential oils can interfere with the growth of microorganisms, mainly by disrupting their lipid cell membrane [24]. Essential oils usually have complex and particular composition that allows them to act in different ways on a broad range of food pathogens, e.g., Gram positive (G+) or Gram negative (G−) bacteria, molds, etc. [25,26]. The most antimicrobial active compounds in essential oils are oxygenated terpenoids, aldehydes, and phenols [27]. Clove [28], thyme [29], rosemary [30], cinnamon [31], cumin [32], oregano [33], and citrus fruit [34], etc., are among the most used plants to obtain essential oils or vegetal extracts for application in antimicrobial food packaging [35].

The hydrophobic nature of essential oils could be considered both an advantage and a drawback at the same time, since this affects the solubility in aqueous systems in which most natural polymers are soluble, limiting the incorporation possibilities into packaging materials [36]. High volatility also limits the quantitative fixation of essential oils into polymeric matrices, leading to poor reproducibility in preparation stages. Moreover, interactions between food and essential oils may occur, which can further reduce activity when tested on food compared with in vitro tests on isolated microorganisms [37].

While the bioactive agents impart functionalities to the packaging materials, the substrate or the matrix has an important role in retaining the bioactive compounds through specific mechanical or physicochemical interactions so that these will be released in a controlled manner [38,39]. The active compounds can be incorporated into packaging materials through various methods, including direct addition into a matrix, deposition onto substrate and incorporation in coatings to induce suitable functionalities [40,41]. Polymers are the most common materials for food packaging due to their versatility, light weight, and low production costs. However, the fossil-based origin of traditional polymers and their long-term persistence in the environment when they end up as waste has raised great concern and required shifting towards new polymeric materials from renewable, natural resources, and/or those with suitable biodegradability [42]. Polysaccharides and proteins are natural polymers extensively applied as edible and biodegradable materials [43], especially for films and food coatings, due to their suitable mechanical and barrier properties [44,45]. Besides, bio-derived or fossil-based synthetic polymers with biodegradability are also largely considered in developing active materials for food packaging. In this category, bio-derived poly(lactic acid)–PLA and poly(hydroxyalkanoates)-PHA [46], and biodegradable synthetic poly(vinyl alcohol)–PVA [47], all of them being FDA-approved as food contact materials [48], are the most used matrices for incorporation of bioactive compounds.

Several natural and biodegradable polymers used in food packaging materials, such as chitosan, alginate, and gelatin, also present stimuli responsive behavior; namely, they are capable of altering their physicochemical properties upon exposure to external stimuli from surrounding media [49]. For example, changes in color, conformation, morphology, and electrical charge can occur under influence of physical or chemical factors such as light, temperature, pH, ionic strength, etc. This behavior can be exploited as an indicator for the freshness or safety of the products or to trigger the release of active agents previously trapped in the polymer matrix. This class represents an attractive category of food packaging and is called “smart” or responsive packaging [50].

## 3. General Aspects of Stabilization Techniques

The overall aim of the incorporation/encapsulation strategies in active packaging is to ensure the availability for a large period of time of a greater amount of the bioactive compounds in the final material. Since natural compounds have large variation in composition and properties, one cannot establish general stabilization methods that could suit a broad spectrum of materials. The selection of an incorporation method usually is particular, depending both on the properties of the bioactive components and of the embedding materials, and also on the targeted food.

**Figure 1:**
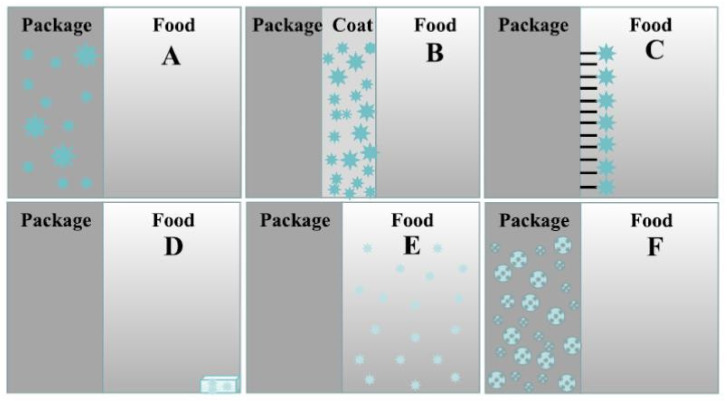
Schematic representation of various methods for incorporation of essential oils into packaging materials: (**A**) direct addition into packaging material; (**B**) incorporation into coatings; (**C**) immobilization onto substrates; (**D**) entrapment into physical carriers; (**E**) addition into headspace; (**F**) incorporation into matrices through carriers. Reprinted with permission from [17]; Copyright 2021 Elsevier.

Fixation usually involves physicochemical interactions between components in liquid phase (melt and especially in solution) by various processes such as melt processing, emulsification, coacervation, molecular inclusion in carriers, crosslinking, or electrospraying/electrospinning, and the final material can be converted in solid form, e.g., by cooling, solvent evaporation, precipitation, etc. [51,52]. The incorporation of essential oils into packaging materials can be usually performed by (i) direct addition to the polymeric materials in earlier steps of material preparation; (ii) adsorption or coating onto support materials; (iii) immobilization onto the surface of the packaging, (iv) mechanical entrapment into physical carriers as accessories for food packaging; (v) introduction into headspace; (vi) microencapsulation in carriers followed by incorporation into matrices [17], as graphically shown in Figure 1.

Direct addition of bioactive compounds can lead to degradation in melts or to weak fusion with the embedding macromolecules in solutions, leading to materials with poor performance. There are various methods to improve the fixation of bioactive agents in a more advantageous way. In this context, emulsification and electrospinning/electrospraying are largely used methods to overcome these limitations by enhancing the stability while maintaining the function of essential oils. Thus, this review focuses on the latest reports on these two techniques. In some cases, for example when the bioactive agents are susceptible to various degradation factors, such as light, temperature, oxidative agents, pH, etc., a prior stabilization of bioactive agents is necessary before incorporation into a packaging material, in order to enhance protection and to limit undesired side-effects by ensuring controlled release. In the particular case of essential oils, these refer to volatilization and alteration of organoleptic properties of food [53,54].

## 4. Fixation by Emulsion of Bioactive Principles

Most natural polymers used in obtaining films or coatings for food active packaging have a hydrophilic nature, which strongly restricts the direct loading with essential oils that, by their nature, are hydrophobic. To overcome this limitation and to widen the applicability of the natural hydrophobic bioactive compounds in active food packaging, emulsification started to be addressed [55]. Emulsion techniques have owned over time an important place in the food industry but lately gained tremendous attention in active food packaging as an encapsulating method. Emulsification represents an attractive strategy to enhance stability and dispersion and to limit degradation, hence ensuring increased and prolonged functionality of essential oils in aqueous systems [56]. Usually, various biopolymers are used to improve the stability and to form coatings or particles [57]. An emulsion represents a colloidal system consisting of at least two immiscible liquids; the oil, which is a property of main interest in food packaging materials, and the aqueous phase, wherein one liquid (the continuous phase) includes a dispersion of droplets of the other liquid (the dispersed phase) [58]. Based on the size order of the droplets, emulsions are categorized into three classes, namely: (i) conventional emulsions, comprising droplets with diameters greater than 1000 nm, (ii) nano-emulsions (NE), which include drops with diameters from 20–1000 nm, and (iii) micro-emulsions, droplets with sizes in the 5–100 nm range [59]. The conventional emulsions are prone to phase separation, droplets coalescence and Ostwald ripening, as a consequence of thermodynamic instability [60]. The ranges of emulsion droplets’ size are not well established at the moment for various classes of emulsions, especially in the case of nano-emulsions, where significant variations of the size range are found [61,62]. The differences among the nano- and micro-emulsions are important to highlight, given that the droplet size range reported overlaps most of the time, in the absence of clearly defined dimension-based boundary values. The major difference is thermodynamic stability, nano-emulsions being more sensitive to external factors compared with micro-emulsions. This stability in micro-emulsions is ensured by the fact that the free energy associated with the colloidal dispersions is lower than the free energy related to the phase separation [63]. Even if in the case of nano-emulsions, the process activation energy has positive values (ΔG* > 0), meaning thermodynamic instability; they are considered metastable systems due to their kinetic stability. Nevertheless, in a particular thermal regime and specific composition nano-emulsions can remain stable for prolonged periods of time [59].

Generally, a third component, such as a surfactant or a texture modifier, which locates at the immiscible liquid/liquid interface, is incorporated to stabilize the emulsion system [64]. Surfactants are compounds that contain in their structure both polar (with affinity for the aqueous phase) and non-polar (or lipophilic) parts, which contribute to minimization (lower) of the interfacial tension between the two phases. The hydrophilic-lipophilic balance (HLB) of the surfactants, which represents an arbitrary scale of the measure of the affinity degree of a compound for water or oil, will dictate which phase will be continuous and which one will be disperse. The selection of the surfactant will depend on the type of the emulsion intended to be obtained. Namely, if a surfactant has a HLB value lower than 6 it will be suitable for obtaining water-in-oil (W/O) emulsions, while higher values of HLB in the arbitrary 6–20 range will indicate a facile dispersion of the oil into aqueous phase, namely oil-in-water (O/W) emulsions [65].

Various studies have shown that decreasing the size of the oil droplets in emulsions towards the nanometric domain will improve their carrier function, which will be reflected in a superior solubility and stability [66]. However, physical instability of nano-emulsions usually occurs during the preparation of film-forming solutions and over the storage period of the film, which manifests by the migration of the oil droplets towards the surface of film [67]. Studies have revealed that solid fine particles have more potential to stabilize emulsions when compared with classical emulsifiers [45,68]. The emulsions that are stabilized by solid particles instead of common stabilizers (surfactants or emulsifiers) are defined as Pickering emulsions [69]. The solid particles stabilize the emulsions through a mechanism similar to those of surfactants, namely by lowering the surface tension between the oil and aqueous phases, but in a more efficient manner. The decrease in interfacial energy happens due to the localization of the solid particles at the interface between the two immiscible phases, which further impedes the droplets’ flocculation and coalescence [58]. The densely packed layer around the emulsion droplets ensures a steric repulsion between them, which translates into better physical stability of the Pickering emulsions when compared with those of conventional ones [70]. The type of the obtained Pickering emulsion, namely O/W or W/O, is determined by the degree of hydrophilicity or hydrophobicity of the particles [71]. Apart from stabilization, the layer of solid particles formed around the essential oil droplets also has the role of carrier and protection against various factors from the surrounding medium (mainly oxidative processes) [72].

The emulsion technique is used in food packaging mainly as a loading method for the development of active materials in the form of capsules, films, coatings, and emulsified gels. Thus, the obtained materials aim, besides the abovementioned functions, to ensure the release of loaded bioactive components in a controlled manner.

Currently there is a growing interest in the development of active food packaging materials based on renewable and environmentally friendly polymers [73]. In the field of bioactive materials containing sensitive essential oils as natural active components, the water soluble polymers have become promising candidates for replacing solvent-soluble and thermal processed materials, since natural bioactive oils have high volatility or are prone to thermal degradation [74].

Polysaccharides and proteins are among the natural biopolymers which have been involved in the development of active materials by incorporation of plant oils through the emulsification method. Of these, some have been extensively applied, such as chitosan [75] and its derivatives [76], starch [36], pectin [77], cellulose derivatives [78], and gelatin [79]. The abovementioned natural polymers are hydrophilic, thus immiscible with plant oils, therefore the research in this field is oriented towards lowering the interfacial energy between the components and providing stability to the emulsion system [80].

Stabilization of the oil droplets in emulsions by water soluble polymers involves mainly two mechanisms, namely: (i) direct adsorption on the interface, or (ii) interaction trough a surfactant or other biopolymer already present at the droplet surface [70]. The involved mechanism depends on the type of the biopolymer, namely if it is surface active (e.g., proteins) or non-surface active (e.g., most polysaccharides). All proteins are amphipathic, while only few polysaccharides contain non-polar fragments in their structure (e.g., chitosan) [81].

In active food packaging applications, the emulsion casting films are the most common. Until now there has been no study that demonstrates stabilization of an emulsion solely by biopolymer presence, therefore combination with surfactants or additional processing treatments is applied. By analyzing the recent studies in the field (see Table 1) it is noted that in most cases Tween 80 (or polysorbate 80), with a HLB value of 15, is used as a surfactant [82], glycerol is often used as a plasticizer [83], and ultrasonication is applied to lower the droplet size, enhancing the emulsion stability and functionality [84].

**Figure 2:**
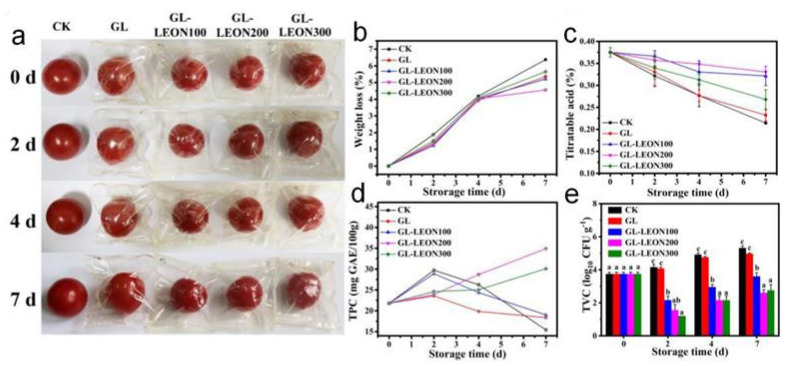
Visual pictures (**a**); changes of weight loss (**b**), titratable acid (**c**), total phenolic content (**d**) and total viable microbial counts (**e**) of cherry tomatoes wrapped by gelatin-lavender essential oil (GL-LEONs) films during storage at 25 ± 2 °C. Different letters at the same storage time (**e**) indicate significant difference. Adapted from [85]; Copyright 2021 Elsevier.

In a study performed by Sun et al. [85], gelatin-based films loaded with lavender essential oil nano-emulsions (LEON) were prepared by solvent casting and further tested for preservation of cherry tomatoes. Significant interactions, mainly through hydrogen bonds between lavender essential oil, Tween 80, and gelatin, were evidenced by infrared spectroscopy. These interactions further induced enhanced hydrophilicity of the materials, which seems unexpected considering the hydrophobic nature of the essential oil. The authors assigned this outcome to the Tween 80 layer formed at the oil droplet surface. LEON incorporation leads to an increased water vapor permeability of the gelatin films, which is assigned to the hydrophobic nature of the oil, causing the weakening of hydrogen bond interactions between the gelatin molecules. The gelatin-based films proved to have a fine heat-sealing property, preventing dehydration, decreasing the weight loss and the titratable acid, increasing the total phenolic content, and imparting antimicrobial activity, hence preserving the freshness and extending the shelf life of cherry tomatoes (Figure 2a–e).

Increased hydrophilicity by incorporation of hydrophobic substances into a hydrophilic matrix was also described by Stoleru et al. [74] when embedding clove essential oil (CEO) and argan vegetal oil into chitosan-based coatings. This feature was attributed to surface enrichment with the hydrophilic fragments of micelles formed with Tween 80 and amphiphilic chitosan.

The addition of proteins can cause thickening and gelling of the watery phase of emulsion under the action of various factors such as temperature (e.g., denaturation of whey proteins [86]) or pH (e.g., gelation of caseinate [87]). Such processing treatments create particular materials with solid-like mechanical characteristics [88], referred to as emulsified gels, or emulgels (EG), which protect and stabilize the oil droplets via the formed gel network [89]. Besides protecting oil droplets, these emulsion gels prevent protein aggregation and thermal denaturation [90]. Due to these peculiarities emulgels have received considerable interest in the past decade in active food packaging-related applications [91,92].

To achieve a better protection of sensitive bioactive oils, a pre-encapsulation stage is often involved, which mainly implies loading oil into capsules. This strategy was applied by Jiang et al. [93] to obtain bioactive composite films based on grass carp collagen and chitosan nanoparticles loaded with lemon essential oil. Nanoparticles were obtained by crosslinking with sodium tripolyphosphate of the lemon EO-chitosan emulsion. Thus prepared nanoparticles were incorporated into grass carp collagen films, which were further evaluated for the inhibition of pork oxidation. The edible films exhibited great oxygen permeability and UV–Vis barrier properties and extended the shelf-life of chilled pork by up to 21 days.

Current evidence on the topic of active food packaging based on emulsions shows that the main interest is directed towards the development of new compositions and few researches have also considered technology tuning.

**Figure 3:**
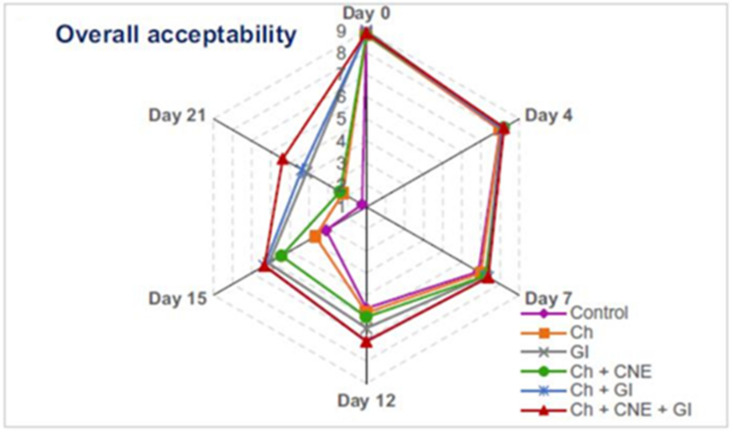
Changes in overall acceptability (based on appearance, odor, and texture) of beef loins treated with chitosan (Ch), cumin essential oil nano-emulsion (CNE), and gamma irradiation (GI) during chilled storage. Adapted from [94]; Copyright 2021 Elsevier.

Dini et al. have combined active chitosan film containing cumin essential oil and low-dose gamma irradiation in preservation of beef loins. Figure 3 shows the overall acceptability, which includes the appearance, odor, and texture of beef loins, and reveals that the treatment which included bioactive chitosan films and gamma irradiation manifested the best efficiency in controlling the microbial flora and inoculated food pathogens and enhanced the shelf life of beef loins (extended for at least 14 days) [94].

Among the polysaccharides, chitosan is extensively used in obtaining emulsion-based systems due to its versatile features, namely its amphiphilic character, cationic nature, film forming ability, biocompatibility, and antimicrobial properties. Because of its wide range of properties, it can take various roles in emulsion systems. Until now chitosan was used as the aqueous continuous phase stabilizing agent, protective layer in capsules/particles, and as a solid particle emulsifier [82].

## 5. Encapsulation of Active Principles by Electrospinning

Electrospinning is a non-mechanical processing technique that uses a high-voltage electrostatic field to electrohydrodynamically stretch droplets from melts and especially from solutions into single, continuous, and very fine jet, at nanometric up to several micrometers scale, which solidifies as self-assembling threads to form nonwoven mats of entangled long fibers [114,115]. This is a simple, versatile, flexible, cost effective continuous process that can be easily upgraded to industrial scale for production of nanofibers with controlled diameter and tailored properties. Rounded or flattened, smooth or highly porous, hollow, core-shell, multilayer coaxial or Janus nanofibers with hydrophilic or hydrophobic character can be obtained, all being characterized by fine size and high aspect ratio, large specific surface area, and superior mechanical properties [116].

Electrospinning of single polymers as well as of polymer-based blends and composites into nanofibers is increasingly considered for applications in food packaging due to several advantages lacking in conventional films and sheets. The types of polymers used for electrospun fibers and the morphology and active properties of the obtained materials intended for food packaging applications were comprehensively reviewed by Topuz and Uyar [117]. Since the electrospinning process is conducted at room temperature, without the need of heating usually involved in the preparation of classical packaging materials, it can be used for inclusion and protection of volatile or thermally sensitive additives, such as essential oils or their components [118]. The high surface area to volume ratio of nanofibers makes them sensitive to external factors; for example, temperature, relative humidity, and pH. This can be exploited for stimuli-responsive systems from which the embedded active agents have tunable release [119]. Annealing of electrospun mats induces fiber coalescence into a continuous layer of transparent film with strong mechanical properties and little porosity [120,121]. Active fibers can be deposited by electrospinning, with increased adherence, onto polymeric supports whose bulk performance (e.g., mechanical properties) is to be maintained and only surface functionalization is required [122,123]. Electrospinning can be also used to induce good adhesion between materials with opposite characters (e.g., hydrophilic proteins and hydrophobic polyesters) that form multi-layered systems [124,125].

Application of electrospinning in food packaging is very limited compared with the strongly increasing use of this technique in various domains such as drug delivery, wound dressing and repairing, textiles, biosensors, electronics, filtration, energy conversion, and storage. One constraint comes from the necessity of using food grade polymers that should be GRAS or FDA-approved. Proper polymer dissolution often requires use of toxic solvents, which are of great concern in food-related applications. The recent trends in overcoming the plastic waste problem also require the shift from classic, fossil-based inert polymers to biodegradable materials originating from renewable or recoverable resources. This strongly limits the range of polymers and additives that can be used. Besides, not all polymers are suitable for electrospinning. Proteins, for example, have intricate secondary and tertiary structure due to strong interactions between the macromolecular chains, which do not allow proper elongation and orientation in continuous threads under the electrospinning conditions [126]. Therefore, suitable solvents are needed to dissociate and unfold proteins in random coil conformation with necessary chain entanglements for electrospinning [127]. Carbohydrates, also, can have unfavorable chain entanglements or chemical structures that limit electrospinnability. For example, the polyelectrolyte behavior of chitosan in acidic solutions leads to repulsions between the ionic groups on the macromolecular chains, resulting in droplets formation instead of continuous fibers [128]. Carrier polymers are therefore used to provide the necessary functionalities for the electrospinning of natural biopolymers, as described in a review of Kakoria and Sinha-Ray [129] focused on biopolymer macro- and nanofibers obtained by electrospinning and solution blowing. Detailed insights on various aspects related to the proteins and carbohydrates used for electrospinning are presented in a comprehensive review by Kumar et al. [123]. Electrospinning of natural polymers for fixation of antimicrobial agents into ultra-thin membranes was reviewed by Rodríguez-Sánchez et al. [130], who also included discussions on analysis techniques to determine the properties of obtained materials.

Since the basic principle of electrospinning consists in overcoming the surface tension of a viscous solution by a high-voltage electrostatic field to generate a continuous jet, the properties of electrospun fibers strongly depend both on the properties of the starting solution (e.g., molecular weight and ionic character of polymer, viscosity, solvent, surface tension, electric conductivity) and on the processing parameters (the applied field strength, solution feeding rate, the distance between the tip and the collector, ambient temperature and relative humidity) [128]. Polymer solutions with too low viscosity cannot sustain continuous jet and flow as droplets, leading to electrospray (electrohydrodynamic atomization). This can be used for encapsulation of bioactive compounds into nanoparticles [131] or for film deposition onto non-flat surfaces [132].

**Figure 4:**
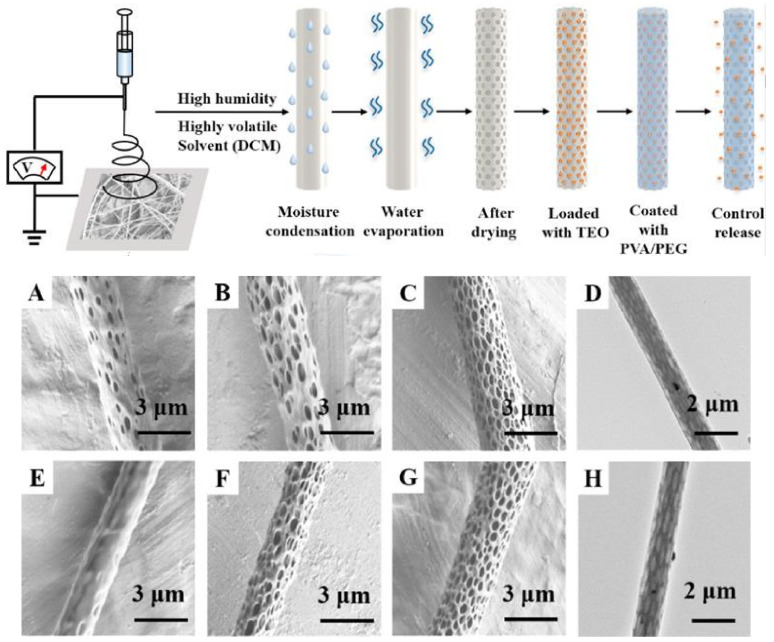
Pictorial visualization of the processes involved in pore formation of electrospun fibers and the SEM images of the obtained fibers showing the effect of dichloroethane/ethanol solvent ratio (90/10, 95/5, 100/0–(**A**–**C**)) and of ambient humidity (20%, 50%, 80%-(**E**–**G**)); TEM images of fibers (**D**,**H**). Adapted from [137]; copyright 2021, Elsevier.

Besides controlling the viscosity and surface tension of the solution, and the morphology of polymeric macromolecular chains, solvents and their volatility also have an important role in the porosity of electrospun fibers. Phase separation phenomena can be induced using mixtures of solvents with different boiling points or solvent/non-solvent systems, leading to various pore structures through electrospinning [133,134]. Bioactive components can be loaded into the porous structure of the nanofibers for protection and controlled release. A schematic representation of pore formation on the electrospun fibers followed by loading with bioactive principle and coating with PVA/poly(ethylene glycol) (PVA/PEG) for controlled release, and the SEM images of porous nanofibers, is presented in Figure 4. The overall porosity of the electrospun mat, shown by the empty spaces between fibers, is strongly affected by the collector geometry, which determines the distribution of the electrical field in which the threads fly from the region of whipping instability at the needle, orienting and aligning the fibers [135]. Solidification of polymers by solvent evaporation occurs very fast during electrospinning, decreasing the glass transition of the material [136].

Emulsification is often involved when components with opposite nature (e.g., hydrophobic essential or vegetal oils and hydrophilic natural polymers) have to be combined for tailoring the final properties of materials. Core-shell fibers can be obtained by electrospinning from a single nozzle the droplets that contain the minor component of water-in-oil (W/O) or oil-in-water (O/W) emulsions, moving inward due to rapid evaporation of the solvent from the outer layers. Details of this phenomenon are discussed in a review of Zhang et al. [138] focused on emulsion electrospinning of food-grade materials. Surfactants used in emulsions are involved in interactions with the components of the system, influencing the interfacial tension and viscosity, with strong effects on electrospinning behavior and the properties of obtained fibers. For example, the release of curcumin from gelatin nanofibers is inhibited by the anionic sodium dodecyl sulfate (SDS) but promoted by non-ionic Tween 80 and cationic sodium cetyltrimethyl ammonium bromide (CTAB) surfactants [139]. At the opposite side, the release of cinnamon essential oil from a zein fiber network was reduced by the cationic CTAB, which affects the secondary structure of zein, but was enhanced by Tween 80 and the anionic SDS [140].

Electrospinning is increasingly used for encapsulation of essential and vegetal oils or their principal constituents into nanofibers of biodegradable polymers for use as food packaging materials. The main methods to encapsulate essential oils into active films were reviewed by Zhang et al. [141], with a section dedicated to electrospinning, while the general aspects related to the electrospinning of essential oils was reviewed by Mele [142], with focus on cinnamon, oregano, peppermint, clove, thyme, and lavender as the most commonly used essential oils. The types of polymers, inorganic fillers, and active substances used in electrospinning to obtain functional materials suitable for food packaging were reviewed by Zhao et al. [143].

Here we collected the most recent scientific works on using electrospinning to encapsulate essential oils into matrices of biodegradable polymers. Table 2 presents the system (polymeric matrix and solvent, active principle, preparation steps), procedure (electrospinning (espin) or electrospraying (espray)), process parameters (flow rate, needle size in Birmingham gauge G system, voltage, tip-to-collector distance, temperature, relative humidity) and the size of obtained fibers or films, food, or food simulant on which the material was tested, and the antioxidant and antimicrobial evaluations, including the particularities of the system.

Some general observations can be drawn from the collected data in Table 2. Most reports considered direct addition of essential oils into polymeric systems before electrospinning, while in some works only the polymeric materials were electrospun to obtain nanofibers or coatings that were afterwards loaded with the low molecular bioactive principle. While direct addition has the advantage of being a one-step process and of incorporating the entire amount of the bioactive compound into the polymeric system, this might affect the electrospinning behavior or the properties of obtained materials, especially when various solvents are needed; for example, when there is a difference in the hydrophobic/hydrophilic nature of the additive/polymeric matrix. On the other hand, loading the bioactive compound after the electrospinning process requires higher amounts of reagents and additional processing steps but has lower impact on the physical properties of the material. Some other studies considered carriers for encapsulation and protection of active agents inside the polymeric matrix. The carrier system including the active principle can be obtained by electrospinning/electrospraying then added to the polymeric matrix. For example, β-ciclodextrins were often used as carriers for essential oils, using two main procedures. One approach considers direct addition of cyclodextrins and of essential oils to the solution of the polymeric matrix, followed by electrospinning. Another method involves a two-step process in which inclusion complexes of β-cyclodextrins and essential oils are firstly obtained (usually involving sonication) then added to the polymeric solutions for further processing by electrospinning. The use of nanophytosomes as a carrier for cinnamon essential oil was also reported [161].

Post treatments such as conditioning [161], chemical crosslinking and physical welding [162] or annealing [146,157], were used in several studies to improve the properties of the electrospun fibers. These can induce crystallization of components, provide better adhesion of electrospun fibers onto base film, slow the release of the active compounds or convert the electrospun fiber mats into films. Nitrogen cold plasma was used by Lin et al. [154] as post treatment for the electrospun fibers of silk loaded with thyme EO. Poly(ethylene oxide) was added to the aqueous solution of silk fibrinoid to increase the viscosity up to levels suitable for electrospinning. Plasma treatment did not change the chemical composition of fibers but induced surface modifications that enhanced the antibacterial activity against *Salmonella Typhimurium*, with stable positive action on poultry meat stored for 7 days.

A work reported by Figueroa-Lopez et al. [146] advanced to the pre-production level of multilayer films in a Fluidnatek^®^ LE500 pilot scale electrospinning equipment with 24 emitters that scan the surface of the collector. A commercial YPACK210 food grade biodegradable film with a 50% content of PHA was coated with a layer of cellulose nanocrystals after corona discharge and addition of Loctite LIOFOL PR1550 food contact primer. A PHVB solution 8% in chloroform/butanol 75/25 containing 2.5% oregano EO and 2.25% ZnO nanoparticles was electrospun onto the surface then an assembly was made with another YPACK210 film to form multi-layered structures with thickness of 130–150 µm. The mechanical performance was slightly decreased but remained balanced while the barrier properties improved due to the cellulose nanocrystal coating, at the expense of lower antioxidant activity due to slower migration of EO. Significant antibacterial effect was observed against both Gram positive *S. aureus* and Gram negative *E. coli* while no cytotoxicity was observed in Caco-2 cells.

Research focuses mainly on aspects that generate the properties of the starting solution by varying the solvent, the surfactant, or other additives, the ratio between components, the procedures for putting the components together (e.g., homogenization and dispersion). These, indeed, provide the main functionality of the final products and determine the overall electrospinning behavior. On the other side, the electrospun processing parameters are usually varied mainly to determine suitable conditions for good fiber formation, and then the active components and related additives are added. However, the changes in composition affect the properties of the solutions and, consequently, the electrospinning behavior, but the fine tuning of processing parameters is usually not considered, even though this can have a strong impact on the functionality of the final product, such as the controlled release behavior.

Additionally, studies have not always been performed in a controlled atmosphere (temperature and humidity), which could have a strong impact on the porosity of the formed fibers. The antimicrobial activity of the final products, which is a property of main interest in food packaging materials, is usually tested on various bacterial strains, with less interest in antioxidant activity. However, when this was considered, it was mainly evaluated based on DPPH assay, and rarely on alternative methods such as ABTS, oxygen radical absorption capacity (ORAC), or ferric reducing antioxidant power (FRAP), which might evidence different mechanisms of action [158]. Few studies have also tested bactericidal activity and cytotoxicity.

## 6. Conclusions and Future Perspectives

In the case of packaging systems obtained by emulsification, a unified trend was noted in preparation procedures, namely most research has involved the use of Tween 80 as surfactant, glycerol as plasticizer, and ultrasonication to lower the droplet size. The polymeric matrices were mainly aqueous soluble polysaccharides or proteins, hence direct O/W emulsions were obtained, and very few works considered inverse emulsions. Particular and more efficient systems were developed when using solid-particle stabilizers (Pickering emulsions) instead of classical surfactants.

Electrospinning was largely used as a versatile method that allows different approaches for fixation of essential oils, such as direct addition into polymeric systems, loading on electrospun fibers, and encapsulation into carriers before electrospinning. Post treatments were used to improve the performance of fibers or to convert fibrous mats into films. Electrospinning enhanced the bioactivity and induced the controlled release of essential oils due to nanostructuration and increased surface to volume ratio.

Research was generally focused on varying the components and their concentration in studied systems and less on finely tuning the process parameters. Antimicrobial performance of materials was tested mainly in vitro on isolated pathogens, especially on *E. coli* and *S. aureus* bacterial strains, but overall, relatively few studies involved tests on food. Only one work [146] stepped up to the level of pre-production, at pilot plant scale.

The effect of bioactive packaging is usually tested on foods a short time after their preparation and a gradual decrease of functionality is observed with time. While this is not regarded as a problem when related to the rather short shelf life of the food products, it should be, however, considered that large periods of time pass in actual practice between production of packaging and their utilization. Further research is needed on long-term stabilization of active agents into materials during storage of packaging, before utilization. Future research should also consider inclusion of essential oils into stimuli responsive polymeric matrices, able to release the bioactive agents as a response to various physical or chemical factors that affect food freshness or safety.

The use of essential oils in the field of active food packaging is still in its incipient stage, far from the necessary applicability on a larger scale and even further away from the application at industrial level. This is due both to the limiting properties of plant oils, namely susceptibility to thermal, light, and oxidative degradation, volatility, hydrophobic nature, and the difficulty of applying a single technology that ensures the stabilization, incorporation, and controlled release of bioactive compounds. At this time, neither emulsification nor electrospinning is applied on a large, industrial scale as a technique for incorporation of essential or vegetal oils. A possible approach that looks promising is the collaborative use of both methods since emulsification brings advantages in stability and compatibility with the polymeric matrices, while electrospinning presents advantages of nano-structuration and easy scaling up to the industrial level. At this moment few scientific studies are reported that apply both electrospinning and emulsification to incorporate vegetal oils into active food packaging materials. Laboratory studies that focus on exploiting the benefits of both methods and the technology tuning are necessary for further advancement in this field.

## Figures and Tables

**Table 1:** Recent examples of essential oils incorporated into biodegradable packaging materials by emulsification method.

Polymeric Matrix/System	Bioactive Oil/Compounds	Procedure/Form of Material	Evaluated Target Properties/Particularities of the Systems	Type of Tested Food	Ref.
***Conventional emulsion solvent casting***					
Potato starch/apple peel pectin composite	*Zataria multiflora* essential oil (ZMEO) and zirconium oxide nanoparticles	Films obtained by solvent casting; EO pre-encapsulated in whey protein isolate (WPI) microcapsule by emulsification	Antioxidant properties evaluation. The addition of ZMEO to the film increased the moisture content and water vapor permeability (WVP). The release of the EO occurs gradually in 95% ethanol.	Quail meat-increased shelf life	[95]
Sweet potato starch	Oregano essential oil (OEO)	Starch octenylsuccination and films by solvent casting	Antimicrobial activity against *Staphylococcus aureus (S. aureus*, G+*)* and *Escherichia coli (E. coli*, G-). OEO incorporation and octenylsuccination were negatively related to the water content, water solubility, and WVP of resulting films.	-	[96]
Starch	Lemongrass essential oil (LMEO)	Edible films based on glycerol-plasticized cassava starch by continuous casting	Antimicrobial activity. Biodegradation profile in soil of starch was maintained.	-	[36]
Basil seed gum	*Zataria multiflora* essential oil	Film casting	Bactericidal activity against *E. coli* and *Bacillus cereus (B. cereus)*. An increase in the antibacterial activity of ZMEO nano-emulsion was observed by decreasing the droplet size.	Not tested	[97]
Chitosan	Cinnamon essential oil (CiEO)	Edible polyelectrolyte films obtained by solvent casting; octenyl succinic anhydride (OSA) modified gum arabic (GA) used as emulsifier	Antibacterial activity against *S. aureus* and *E. coli*. Water resistance was improved; sustained release of CEO was obtained in 60% glycerol solution; Schiff-base reaction between CS and CEO may determine changes in film color.	Not evaluated	[98]
Chitosan	*Artemisia campestris* hydroalcoholic extract, aqueous extract (ACHE) and essential oil	Films	Antioxidant, metal chelating ability and UV–Vis barrier properties. Improved water resistance; covalent interaction and H-bonding between chitosan and ACHE. Reduced film extensibility.	-	[99]
Chitosan	Thyme essential oil (TEO)	Chitosan-based emulsions made of liposomes loaded with TEO as edible food coating.Liposomal (using lecithin) chitosan emulsions were prepared via reverse-phase evaporation method.	Antimicrobial activities: total bacteria counts of mesophilic, psychotrophic bacteria, yeast and mold. Coatings showed neutral effects on moisture content, pH and titratable acidity of cheese.	Karish cheese	[80]
Chitosan	Basil EO	Emulsion-based food coating; plasticizer–glycerol; emulsifier-Tween 80; beeswax–lipohilic phase; ultrasonication	Antibacterial activity: *E. coli* and *S. aureus*.	Eggs-extended shelf life	[100]
Chitosan	Cumin EO	Films by solvent casting supplementary γ-irradiated (2.5 kGy)Emulsifier-Tween 80; plasticizer–glycerol; ultrasonication	Enumeration of *Listeria monocytogenes (L. monocytogenes), E. coli,* and *Salmonella Typhimurium* (*S. Typhimurium)* and of microbial flora (mesophilic and psychrophilic bacteria, *Enterobacteriaceae*, and lactic acid bacteria).	Beef loins	[94]
Chitosan-gelatin and pectin-gelatin	Lemongrass essential oil, ZnO, and Zn(CH_3_COO)_2_⋅2H_2_O	Casting method of emulsions and dispersions	In vitro antibacterial activity against *E. coli*, *Bacillus subtilis* (*B. subtilis)*, and *S. aureus*. A synergistic effect between LMEO and ZnO or Zn-Ac was observed both in vivo and in vitro.	Fresh raspberries (*Rubus idaeus* L.)-shelf life extension from four to eight days	[79]
Pectin	Oregano essential oil (OEO) and resveratrol (RES)	Nano-emulsion loaded pectin edible coating in high oxygen modified atmosphere packaging	Microbiological evaluation by total viable count. Minimization of the pH and color change, retarding lipid and protein oxidation, maintaining meat tenderness, and inhibiting microbial growth.	Fresh pork loin preservation	[101]
Gelatin	Lavender essential oil	Firstly O/W nano-emulsion was obtained with Tween 80 and added to gelatin/glycerol system to form films casting approach	Antimicrobial (*S. aureus*, *E. coli* and *L. Monocytogenes*). Antioxidant capacity against 2,2-diphenyl-1-picrylhydrazyl (DPPH) and 2,2′-azino-bis(3-ethylbenzothiazoline-6-sulfonic acid) (ABTS) radicals.	Cherry tomatoes	[85]
Sodium alginate	EO of *Rosmarinus officinalis* L., *Artemisia herba alba* Asso, *Ocimum Basilicum* L. and *Mentha pulegium* L.	Edible films by solvent casting	Antimicrobial activity against *S. aureus*, *E. coli*, *Salmonella enterica*, *Enterococcus faecium*, *Klebsiella pneumoniae* and *Enterococcus faecalis*.	Not tested	[102]
Konjac glucomannan/carrageenan	Camellia oil	Edible emulsion coatings	Microbiological evaluation: total viable counts and psychrophilic bacteria counts.	Chicken meat	[103]
***Pickering emulsions***					
Whey protein isolate/nanocellulose	Bergamot oil	O/W emulsion casting/film	Antimicrobial and antioxidant activity against *E. coli, L. monocytogenes, S.aureus* and *Pseudomonas aeruginosa (P. aeruginosa)*.	Not tested	[56]
Whey protein isolate incorporated with chitosan nanofiber	Cinnamon oil (CiEO)	Oil loaded into nano-structured lipid carriers-NLC (cocoa butter and Tween 80) by ultrasonication; nanocomposites films by casting method	Bactericidal activity: *E. coli*, *S. aureus*, and *P. aeruginosa*. Excellent barrier against water, light and UV permeability; plasticizing effect of emulsified and NLC form of EO.	Not evaluated	[82]
Arrowroot starch/cellulose nanocrystals	EOs from *Menthaspicata* and *Cymbopogon martinii* and carnauba wax nano-emulsion	Film casting	In vitro antifungal activity against *Rhizopus stolonifer* and *Botrytis cinerea*	Not tested	[104]
Chitosan	Cold-pressed rosehip seed oil	Films by solvent casting from emulsionsTween 80–emulsifier; Montmorillonite nanoclay C30B–solid stabilizer for emulsion	Antibacterial properties *E. coli, S. typhymurium*, and *B. cereus* Antioxidant: DPPH radical	Not tested	[105]
Chitosan	Clove essential oil	Composites films; halloysite nanotubes were used to stabilize the oil droplets and glycerol as plasticizer.	Antioxidant: DPPH and reducing power assay. Migration evaluation in food simulants: 50% ethanol-simulant for oil-in-water emulsions and alcoholic foods, and 10% and 95% ethanol, which are stimulants of aqueous and fatty foods; release of CEO was fastest when the films were immersed in 50% ethanol.	Sliced bread	[106]
Chitosan	Clove essential oil	Chitosan-based edible films loaded with zein stabilized Pickering emulsion	Antibacterial: *E. coli* and *S. aureus*.	Not tested	[107]
Carboxymethyl cellulose–poly(vinyl alcohol)	CiEO	Pickering emulsion casting	Antioxidant and antifungal *Penicillium digitatum* properties; UV inhibitory effect.	Bread	[78]
Pectin	Marjoram (*Origanum majorana* L.) essential oil	Films obtained by solvent casting of nano-emulsions and Pickering emulsions	Antibacterial activity of emulsions against *S. aureus* and *E. coli*–by well diffusion technique. Antioxidant, controlled release (food simulant 95% ethanol), and water barrier properties for the films.	Not evaluated	[77]
***Addition through carrier***					
PLA	*Cinnamomum cassia* essential oil, eugenol, and linalool	Capsules by emulsion solvent evaporation method	Bactericidal activity against *E. coli, S. aureus, L. monocytogenes,* and *Salmonella*. Linalool had a possible chemical interaction with PLA; noted the plasticizing effect of active compounds; the capsules presented two release stages and sustaining activity against pathogens for up to 28 days.	Not tested	[108]
Pectin	Copaiba oil (CP)	Copaiba oil nano-emulsions (NE) CP-NE were added to film-forming formulations based on pectin and then dried into films by continuous casting	Antimicrobial activity against *S. aureus* and *E. coli*. The nano-emulsions caused increased roughness, gradual reduction of the elastic modulus and tensile strength, and increased elongation at break. The films preserved their biodegradation profile.	Not tested	[109]
Poly(butylene adipate-co-terephthalate) (PBAT) and chitosan	Cinnamon essential oil (CiEO)	Chitosan nanocapsules loaded with CiEO incorporated in PBAT films by casting;nanocapsules prepared by ionic gelation with sodium tripolyphosphate CN-EO	Biological activity inhibition against *E. coli*. Migration study of EO into distilled water, ethanol, or acetic acid medium; 8% CN-EO content improved tensile strength due to the chemical interactions between chitosan and PBAT.	Not evaluated	[110]
Pullulan	Cinnamaldehyde, eugenol, and thymol	Emulsion-doped active films;encapsulation using liquid (refined coconut oil) and solid (hydrogenated palm oil) carrier oils	Antifungal activity against *Rhizopus stolonifer*, *Alternaria* spp., and *Aspergillus niger*. Controlling and reducing postharvest disease.	Not tested	[111]
Pullulan-gelatin	Clove essential oil (CEO)	The CEO loaded nano and Pickering emulsions prepared with Tween 80 and whey protein isolate/inulin mixture, respectively were incorporated into pullulan-gelatin film	Excellent mechanical properties, water barrier properties, and appreciable antioxidant activities. CEO-loaded PE showed slow-release profile in the film sample	-	[112]
Gellan gum-chitosan complex	TEO	Polyelectrolyte gellan gum (GG)-chitosan (CS) multilayer film was fabricated by layer-by-layer assembly technology with the incorporation of TEO coarse emulsion (TEOC) or nano-emulsion (TEON)	Antimicrobial activity against *E. coli* populations in liquid model system. Films incorporated with TEON showed improved mechanical flexibility and UV blocking property in comparison to TEOC.	-	[113]
Grass carp collagen-chitosan	Lemon essential oil (LEO)	Chitosan (CS)-LEO nanoparticles were prepared by emulsification using ionic gelation with sodium tripolyphosphate;films loaded with chitosan-LEO nanoparticles were obtained	Microbial analysis and sensory evaluation. Great preservative and antioxidant efficacy for 21 days. The LEO release rate increased with decreasing GCC:CS-LEO ratio.	Fresh pork meat	[93]
***Physical adsorption/impregnation***					
Cellulosic pads	Thyme and oregano essential oils	Emulsion adsorption	Antimicrobial reduction of the psychrophilic microbiota.	Minced beef	[11]

**Table 2:** Recent examples of essential oils or vegetal oils incorporated by electrospinning (espin)/electrospraying (espray) into polymeric materials for food packaging.

System	Procedure/Form of Material/Size	Food/Food Simulant	Antioxidant/Antimicrobial Evaluations; Particularities	Ref.
***Loading into matrix***				
CaCl_2_ crosslinked carboxymethyl chitosan film by espray from ethanol 50%, then loaded with carvacrol nano-emulsion in H_2_O + surfactants	- espray: 24G, 0.1 mL/min, 25 kV, 10 cm, 25 °C - 9–30 µm, films	- bread	DPPH - *S. aureus* - *E. coli*	[144]
PLA espin fibers from dichloromethane/ethanol, then loaded with thyme EO in ethanol 50%, then coated with PVA/PEG in water	- espin: 2 mL/h, 20, 50, 80 RH - 200–450 nm, fibers	- strawberries	- *E. coli* - *S. aureus*	[137]
***Direct addition***				
chitosan 1% in formic/acetic acid 1/1 + PCL 6% in formic/acetic acid 1/1 + oregano EO (1, 3, 5%)	- espin: 0.1 mL/h, 18 kV, 23G, 15 cm - 110–470 nm, fibers		- *S. aureus* - *L. monocytogene* - *Salmonella enteritidis* - *E. coli* - H bonds between EO and CO groups in PCL	[145]
poly(hydroxy valerate-co-hydroxy butyrate) (PHVB) 10% in chloroform/butanol 75/25 + 1% oregano EO, rosemary extract or green tea extract; annealing of fibers	- espin: rol-to-rol, 18G, 24 emitters, 4 mL/h/emitter, 38 kV dual polarization, 25 °C, 40 RH, 20 cm vertical - annealing: 125 °C, 15 s - 60–80 µm, films		- DPPH - *S. aureus* - *E. coli*	[146]
PHVB 10% in chloroform/butanol 75/25+ oregano EO + ZnO nanoparticles	- espin: rol-to-rol, 18G, 24 emitters, 6 mL/h/emitter, 17 kV, 20 cm vertical - annealing: 125 °C, 15 s - 80–130 µm, films	- ethanol 10% - acetic acid 3%	- *S. aureus* - *E. coli*	[147]
PHA + PHBV 8% in chloroform/butanol 75/25 + 2.5% oregano EO + 2.25% ZnO NP, espun onto YPACK210 film (50% PHA) coated or not with cellulose nanocrystals	- espin: rol-to-rol, 18G, 24 emitters, 6 mL/h/emitter, 18.5 kV, 25 cm vertical - 130–150 µm, multilayer films	- ethanol 10% - acetic acid 3% - olive oil	- DPPH - *S. aureus* - *E. coli* - no citotoxicity for Caco-2 cells	[148]
zein 22% in ethanol 80% + κ-carrageenan 1% in water + ZnO NP + rosemary EO	- espin: 1 mL/h, 21G, 15 kV, 10 cm, 25 °C, 30 RH - 670 ± 240 nm, fibers		- DPPH - *S. aureus* - *E. coli* - H bonds (amino in zein, OH in κ-carrageenan)	[149]
gelatin 12% in acetic acid 88% + angelica EO, 3, 6, 9%	- espin: 23G, 0.3 mL/h, 15 kV, 20 cm - 150–550 nm, fibers		- DPPH - *S. aureus* - *E. coli* - biocompatible for NIH-3T3 cells	[150]
zein 20% in ethanol 70% + 1% cinnamaldehyde + surfactant (T80, SDS, CTAB, lecithin; 2, 4, 6%)	- espin: 18G, 0.3 mL/h, 13–15 kV, 12 cm, 30 °C, 50 RH - 135–300 nm, fibers	- mushrooms - migration in ethanol 10%	- surfactants changes conductivity and fiber shape - release retarded by CTAB, enhanced by T80, SDS, lecithin	[140]
kafirin 0.3–0.4 g/mL in glacial acetic acid or acetic acid/butanol 75/25 + thymol or carvacrol 0.01, 0.05, 0.1, 0.2 g/mL	- espin: 20G, 15–20 kV, 15 cm - 180–400 nm, fibers		- thymol/carvacrol increases/decreases the hydrophilicity - H bonds converts β-turn structures into β-sheets	[151]
guar gum 0.5% in water + PLA 15% in ethanol/acetic acid 1/1 + thyme EO 10, 30%	- espin: 21G, 1 mL/h, 14–16 kV, 25 °C, 30 RH - 414 ± 169 nm, fibers		- DPPH - *S. areus* - *E. coli*	[152]
zein + ethyl cellulose 20% in ethanol 80% +T80 + 1% CiEO	- espin: 18G, 0,005 mL/min, 13 cm, 10–13 kV, 30 °C, 50 RH - 190–430 nm, fibers	- mushrooms		[153]
silk fibroin + poly(ethylene oxide) in water + thyme EO; N_2_ cold plasma treatment of espun fibers	- espin: 22G, 1.0 mL/h, 18 kV, 15 cm, 25–35 RH - 150–300 nm, fibers	- poultry meat (chicken, duck)	- *S. Typhimurium* - high release and antibacterial activity (stable for 7 days)	[154]
chitosan 1.5% in acetic acid 1% + 1.5% poly(ethylene oxide) + chrysanthemum EO	- espin: 21G, 0.2 mL/h, 25 kV, 15 cm, - 50–250 nm, fibers	- beef - ethanol	- *L.monocytogenes* - inhibition rate > 99% at 4–25 °C after 7 days	[155]
chitosan + flaxseed mucilage in ethanol/acetic acid/water 50/45/5 + *Ziziphora clinopodioides* EO + sesame VO	- espin: 23G, 2 mL/h, 15 kV, 25 cm, 25 °C, 30–40 RH - 135–285 nm, fibers	- phosphate buffered saline, pH 7.4	- DPPH - *S. aureus* - *L. monocytogenes* - *S. typhimurium* - *E. coli*	[156]
denaturated soy protein 11% in water + PVA 11% in water + Triton™ x-100 surfactant + β-carotene in soybean oil, espun onto poly(hydroy butyrate) PHB/PHBV film; annealing to promote PLA crystallization	- espin: 0.15 mL/h, 18 kV, 20 cm onto PHB/PHBV film - annealing: hot press <200 °C, 1 min	- soybean oil	- annealing provides better adhesion of espun fibers onto base film and slower the release of active principle	[157]
poly(ε-caprolactone) 20% in 1,2-dichloroethane + α-tocopherol	- espin: 20G, 0.18 mL/min, 15 kV, 15 cm - 1–10 µm, fibers	- curd cheese - ethanol 95%	- DPPH/ABTS	[158]
***Addition through carrier***				
electrosprayed NP of maize zein 10% in ethanol 80% + clove EO 1%, added to potato starch 5g/80 mL in water + glycerol for solvent casting	- espray: 18G, 1 mL/h, 25 kV, 15 cm, 25 °C, 25 RH	- water - ethanol 10, 50%	edible coating	[131]
NP of chitosan 0.5% in acetic acid 1% + T80 +cinnamon EO 2% in ethanol + sodium tripolyphosphate, added to PLA 25% in trichloroacetic acid/dichloromethane 8/2	- espin: 19G, 1 mL/h, 20 kV, 15 cm, 40–50 RH		- *E. coli* - *S. aureus*	[159]
NP of chitosan 0.75% in acetic acid 0.75% + PVA 0.75% in water + SDS + cabreuva EO + sodium citrate, added to PVA in water	- espin: 0.4 mL/h, 24 kV, 27 cm, - 275–363 nm, fibers	- ethanol, water	- *Candida albicans* - *E. coli* - *S. aureus* - *Staphylococcus epidermidis*	[160]
nanophytosome of lecithin + cinnamon EO in ethanol absolute, dried, added to PVA in water + glycerol + boric acid	- espin: 18G, 1.5 mL/h, 17–19 kV, 18 cm vertical, 20 °C, 20 RH - conditioning at 25 °C, 55 RH, 48 h - 31–70 µm, film	- shrimp	- *S. aureus* - *E. coli* - *P. aeruginosa* *no cytotoxicity for HT-29 cells*	[161]
PVA 8% in water + *β*-cyclodextrin + cinnamon EO; fumigation with glutaraldehyde	- espin: 18G, 0.6–0.9 mL/h, 12–15 kV, 15 cm, rotating drum 100–150 rpm, 27 °C, 44 RH	- mushrooms	- *S. aureus* - *E. coli* - simultaneous chemical crosslinking and physical welding by fumigation	[162]
PVA 6–10% in water + *β*-cyclodextrin + cinnamon EO	- espin: 20G, 0.2–0.6 mL/h, 13–17 kV, 12–16 cm, 26 °C, 56 RH - 240 ± 40 nm	- strawberry	- *S. aureus* - *E. coli*	[23]
IC of β- cyclodextrin in water + thyme EO in ethanol, converted to NP with sodium tripolyphosphate and ε-poly(lysine), added to gelatin 25% in acetic acid 20%	- espin: 21G, 0,4 mL/min, 20 kV, 15 cm, 25 °C, 35 RH - 150–230 nm, fibers	- chicken (immersed in *Campylobacter jejuni* suspension)	- *Campylobacter jejuni*	[163]
IC of *β*-cyclodextrin in ethanol 33% + cinnamon EO in ethanol, added to PLA in dichloromethane/N,N-dimethylformamide	- espin: 20G, 2.0–2.4 mL/h, 12–16 kV, 20 °C, 33 RH - 350–1490 nm, fibers	- pork	- *E. coli* - *S. aureus* - higher EO encapsulation by espinning than by solvent casting	[164]
IC of α or γ-cyclodextrin + oregano EO by kneading or freeze drying, added to PHBV 10% in 2,2,2-trifluoroethanol	- kneading/freeze dry - espin: 18G, 24 emitters, 0.6 mL/h/emitter, 18 kV, 20 cm vertical - 0.6–1.35 µm, fibers - annealing: 160 °C, 10 s - 60–86 µm, film		- DPPH - *S. aureus* - *E. coli*	[26]
IC of β-cyclodextrin + *Litsea cubeba* EO, added to dandelion polysaccharide/PEO 2/33% water	- espin: 0.5 mL/h, 17 kV - 241 nm, fibers	- ethanol	- *S. aureus*	[165]
IC of β-cyclodextrin + eucalyptus EO, added to corn zein 30% in ethanol 70%	- espin: 18+ /3-kV dual polarization, 15 cm, 23 °C, 45 RH - 330–390 nm, fibers		- *L. monocytogenes* - *S. aureus* - *B. cereus* - *E. coli* - *S. Typhimurium* - *P. aeruginosa* - *Shigella dysenteriae*	[166]
IC of β-cyclodextrin + gangal root oil, added to swim bladder gelatin 25% in acetic acid 30%	- espin: 0.4 mL/h, 23 kV, 15 cm, - 65–245 nm, fibers	- beef (soaked in *E. coli* suspension)	- *E. coli* (EHEC O157)	[167]

NP: nanoparticles, IC: inclusion complex.
